# GPR21 KO mice demonstrate no resistance to high fat diet induced obesity or improved glucose tolerance

**DOI:** 10.12688/f1000research.7822.2

**Published:** 2016-06-17

**Authors:** Jinghong Wang, Zheng Pan, Helene Baribault, Danny Chui, Caroline Gundel, Murielle Véniant

**Affiliations:** 1 Department of Metabolic Disorders, Amgen Inc., Thousand Oaks, CA, USA

**Keywords:** GPCR, Rabgap1, Diabetes, Drug target, TALENS technology

## Abstract

*Gpr21* KO mice generated with *Gpr21* KO ES cells obtained from Deltagen showed improved glucose tolerance and insulin sensitivity when fed a high fat diet. Further mRNA expression analysis revealed changes in *Rabgap1* levels and raised the possibility that *Rabgap1* gene may have been modified. To assess this hypothesis a new *Gpr21* KO mouse line using TALENS technology was generated. *Gpr21* gene deletion was confirmed by PCR and *Gpr21* and *Rabgap1* mRNA expression levels were determined by RT-PCR. The newly generated *Gpr21* KO mice when fed a normal or high fat diet chow did not maintain their improved metabolic phenotype. In conclusion, *Rabgap1* disturbance mRNA expression levels may have contributed to the phenotype of the originally designed *Gpr21* KO mice.

## Introduction

The G-protein receptors (GPCRs) are the largest family of proteins targeted by drug discovery. GPCRs are crucial molecular sensors for many vital physiological processes. GPR21 is part of the GPCRs family and shares 71% identity to GPR52. It was identified along with GPR22 and GPR23 based on their homology to GPR20 ( O’Dowd *et al.*, 1997). Originally, GPR21 was detected in regions of the brain and later, several other tissues as spleen, brown fat, and macrophages, were reported to express high levels of GPR21 mRNA ( Gardner *et al.*, 2012; Osborn *et al.*, 2012). The natural ligand of GPR21 remains unknown; however, constitutive activity of the GPR21 receptor has been observed when it was co-transfected with Gα15/16 proteins in HEK293 cells ( Gardner *et al.*, 2012; Xiao *et al.*, 2008). Also, GPR21 has been reported to activate the Gq pathway on calcium-sensitive CHO cells ( Bresnick *et al.*, 2003).

In *Gpr21* KO mice generated with *Gpr21* KO ES cells obtained from Deltagen (Deltagen GPR21, Deltagen San Mateo, CA), Osborn *et al.* and Gardner *et al.* have reported that glucose tolerance and insulin sensitivity were improved when compared to their wildtype control mice ( Gardner *et al.*, 2012; Osborn *et al.*, 2012). These *Gpr21* KO mice were leaner than their wildtype littermate control (Osborn *et al.*) and were resistant to diet-induced obesity ( Gardner *et al.*, 2012), making GPR21 a potential drug target candidate for the treatment of diabetes and obesity. Reduced inflammation and macrophage infiltration were also observed in the KO mice ( Osborn *et al.*, 2012).

Mouse *Gpr21* gene is located on chromosome 2 within the intron of *Rabgap1* gene, between exon 13 and 14 according to the UCSC GRCm38/mm10 assembly. *Strbp* gene is located on the opposite strand in the same region. Deltagen *Gpr21* KO mice contain a deletion in the gene of exon one with the insertion of a 5.3 kb lacZ/Neo cassette. After considering the location of the insertion of the neo cassette, we hypothesized that the gene structures, the expression and physiological functions of *Rabgap1* and Strbp may have been altered.

In brief, small RAB GTPases are essential for the coordination of vesicle budding, transport, and fusion of vesicles ( Frasa *et al.*, 2012). RAB proteins are activated by guanine nucleotide exchange factors (GEFs) and inactivated by RAB GTPase activating proteins (RABGAPs). The TBC (TRE2-BUB2-CDC16) domain facilitates the RAB GTP hydrolysis from the GTP-bound active form to the GDP-bound inactive form. However, the physiological function of RABGAP1 (TBC1D11) is less understood. It may be implicated in microtubule and Golgi dynamics during cell cycle and regulation of spindle checkpoint ( Cuif *et al.*, 1999; Miserey-Lenkei *et al.*, 2006). STRBP (SPNR) is a microtubule-associated RNA-binding protein localized in developing spermatids and plays an important role in normal spermatogenesis and sperm function ( Pires-daSilva *et al.*, 2001). *Strbp* deficient mice are smaller, have neurological defects, a high premature mortality rate, show reduced fertility and mating drive as well as abnormal sperm motility.

After further analysis of the Deltagen *Gpr21* KO mice, we observed that *Rabgap1* mRNA expression levels were modified. To assess if the metabolic phenotype observed in these KO mice ( Gardner *et al.*, 2012; Osborn *et al.*, 2012) was solely related to knocking out the *Gpr21* gene, we generated a new line of *Gpr21* KO mice using the TALENS technology. We created a 29 bp deletion within the coding exon of *Gpr21* ( *Gpr21* TAL 29bp), a location very close to the ATG, an out of frame mutation and an early termination of *Gpr21*. The phenotypic analysis of our new *Gpr21* TAL 29bp KO mice showed no improvement of the previously observed metabolic parameters that were identified in Deltagen *Gpr21* KO mice. The originally published improved metabolic phenotype of the Deltagen *Gpr21* KO mice was not solely due to the deletion of the *Gpr21* gene, *Rabgap1*may have been implicated.

## Materials and Methods

### Animals and *Gpr21* ( *Gpr21* TAL 29bp) KO mice generation

All animal experiments were approved by the Institutional Animal Care and Use Committee of Amgen. Mice were housed in a pathogen-free facility with a 12 h light-dark cycle at 22°C. Mice were allowed ad libitum access to water and food. Single housed male *Gpr21* KO ( *Gpr21* TAL 29bp) and their male littermate mice were used in this study. Mice were fed a normal chow (Harlan 2920) until they were 11 weeks old and then a high fat diet (Research Diets D12451,45 kcal % fat) for the next 15 weeks.

GPR21 KO mice were created using a pair of transcription activator-like effector nucleases (TALENs) from Life Technologies targeting exon 2 of mouse GPR21. TALEN binding sites are underlined below with a 15 base pair spacer between the 2 sites.

5 ’- TGAACTCCACCTGGGATGG **TAATCAGAGCAGCCA** TCCTTTCTGTCTTCTGGCA

ACTTGAGGTGGACCCTACC **ATTAGTCTCGTCGGT** AGGAAAGACAGAAGACCGT – 5’

Design, cloning and validation of the TALENs were performed by Life Technologies. Messenger RNA (provided from Life Tech) for each of the TALENs were diluted in RNAse free microinjection buffer to a final concentration of 4.0 ng/µl for each TALEN (8.0 ng/µl total concentration). The TALENs were microinjected into the pronucleus of fertilized one-cell embryos (0.5 days post coitus) obtained from the mating of C57BL/6 (Taconic) males to superovulated C57BL/6 (Taconic) female mice. Microinjected eggs were transferred to pseudopregnant Swiss Webster recipients. Founder pups were screened for TALEN induced mutations in GPR21 by sequencing across exon 2. Two founders, one with a 5 bp deletion and the other with a 29 bp deletion were expanded for further analysis.

### Genomic DNA preparation and PCR genotyping

Genomic DNA was prepared from liver, BAT and spleen using DNeasy blood and tissue kit (Qiagen, Valencia, CA) following manufacturer’s instruction. PCR were carried out using the primers 5’-CAGCATGAAGTGAGAGCCAG-3’ and 5’-CAAGTAGCCCAGTGCCAGAAG-3’.

### Microarray and data analysis

Tissue samples from the Deltagen *Gpr21* knockout mice were used for microarray analysis. These samples were collected by Gardner *et al.* during the course of their *Gpr21* knockout mice study. mRNA was isolated from 6 animals for each group using Qiagen RNeasy Mini Kit (Qiagen, Valencia, CA) and processed following the protocols described in section 2 (Eukaryotic Sample and Array Processing; 701024 rev 1) of the Affymetrix Technical manual. Briefly, 5 µg total RNA was used to synthesize cDNA (10 pmol of T7-(dT)24 primer, and Superscript II (Invitrogen, Carlsbad, CA). Purified double-stranded cDNA (MinElute Reaction Cleanup Kit, Qiagen, Valencia, CA) was used to generate biotinylated cRNA using Bioarray HighYield RNA Transcript labeling Kit (Enzo Diagnostics, Farmingdale, NY) followed by purification with Qiagen RNeasy Mini kit and hybridization to the Affymetrix HT MG 430 PM array. Arrays were washed on a GeneChip Fluidic Station 450 (EukGE_WS2v4_450 protocol) and scanned using the Affymetrix GeneChip Scanner 3000 (Affymetrix, Santa Clara, CA). Data analysis was conducted with R (version 2.15, http://r-project.org) with Bioconductor (version 2.10, http://bioconductor.org/) and ArrayStudio (Omicsoft, version 8.0). Briefly, Affymetrix CEL files were normalized in Bioconductor using the GCRMA method. Differentially regulated genes were identified using a moderated t-test. False discovery rate adjusted P-values were calculated using the method of Benjamini and Hochberg.

### RNA isolation and expression assays

Total RNA was isolated using Qiagen midi RNA preparation kit (Qiagen, Valencia, CA). The total RNA concentration was determined with a Nanodrop (ThermoFisher Scientific, Wilmington, DE). QPCR was performed using 10 ng RNA per well, Taqman master mix (Applied Biosystems, Foster City, CA). *Rabgap1* gene expression was assessed using Taqman Probes from Applied Biosystems (Mm01327207_m1 and Mm01327199_m1). Gpr21 mRNA level was measured using the forward primer 5’-CACCTGGGATGGTAATCAGAG-3’, reverse primer 5’- TCACAATGATGTTGCCAGAAAT-3’ and probe 5’

FAM/TTCTGGCAC/Zen/TGGGCTACTTGGAAA/IABkFQ-3’ from Integrated DNA Technologies (Coralville, IA). Results were evaluated using the ΔΔC _T_ method and normalized relative to the expression of glyceraldehyde-3-phosphate dehydrogenase *(Gapdh)*.

### Oral glucose (OGTT) tolerance test

17 *Gpr21* TAL 29bp knockout and 17 wildtype littermate mice were used in this experiment. Glucose, body weight and OGTT were measured on 11 week old mice fed a normal chow diet. Then, mice were switched to high fat diet (HFD) feeding. Two more glucose, body weight and OGTT measurements were performed on 15 and 26 week-old mice (fed HFD for 4 and 15 weeks, respectively). At 6 am, mice were fasted for 4 hr. Glucose levels were measured and blood samples were taken from the tail vein before oral glucose tolerance test (OGTT) was initiated. GTT was performed by oral administration of a bolus glucose (2g/kg body weight). Glucose levels were measured at 20, 40 and 60 min after glucose administration by using AlphaTrak blood glucose meter (Abbott, Chicago, IL).

### Serum insulin measurement

Blood samples collected before OGTT, were centrifuged at 10000rpm. Serum insulin levels were determined by using Insulin (mouse) ultra-sensitive EIA kit 80-INSMSU-E10 or mouse high range insulin ELISA 80-INSMSH-E01 (ALPCO Diagnostics, Salem, NH).

### Statistical analyses

Two-way ANOVA followed by Bonferroni was used to compare more than two groups. For comparison between two groups, unpaired two-tailed t test was performed. All tests used the software GraphPAD Prism (GraphPad, San Diego, CA). Significance was defined as *: *P* < 0.05; **: *P* < 0.01; ***: *P* < 0.001.

## Results and discussion

### *Rabgap1* expression was changed in Deltagen *Gpr21* KO mice

**Figure 1.**
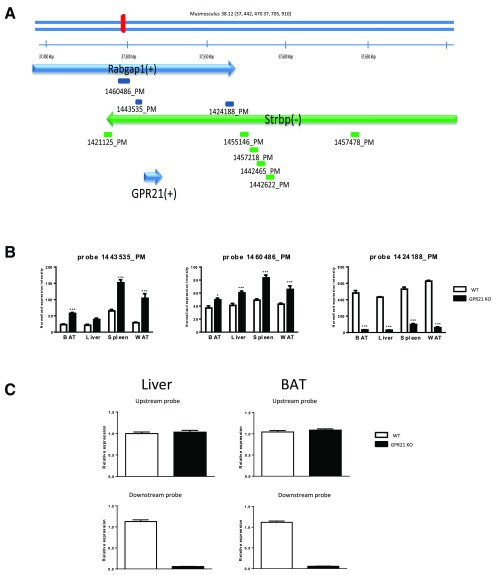
( **A**) Mouse *Gpr21* is located on Chromosome 2 within the intron of *Rabgap1* gene between exon 13 and 14 on the positive strand according to UCSC GRCm38/mm10 assembly. *Strpb* gene is on the opposite strand in the same region. The blue arrow represents the positive strand while the green one the negative strand. The bars under the genes represent microarray probe sets from Affymetrix mouse array HT MG-430PM platform. There is no probe set covering *Gpr21* gene. The closest probe set 1421125_PM is located at 2,866 bases upstream of *Gpr21*. ( **B**) The level of *Rabgap1* transcript was shown as normalized expression intensity. RNA was prepared from BAT, liver, spleen and WAT of Deltagen *Gpr21* KO mice and their WT littermate controls. Probe 1443535_PM, 1460486_PM and 1424188_PM allow detection of *Rabgap1* mRNA expression levels. ( **C**) *Rabgap1* mRNA expression levels were assessed using 2 Taqman probes in liver (left panel) and BAT (right panel) of GPR21 KO and their wildtype littermate mice.

We isolated RNA from spleen, liver, perirenal fat (WAT) and brown fat (BAT) from Deltagen *Gpr21* KO mice and their wildtype littermate control mice. Microarray results identified that *Rabgap1* was the only gene that was changed in all the tissues analyzed. Rabgap1 mRNA levels were increased by 1–4 fold when using two independent *Rabgap1* probes ( Figure 1A, Figure 1B, Table 1) located upstream of *Gpr21* gene and were down regulated by 5–15 fold when using one *Rabgap1* probe located downstream of *Gpr21* gene. This result indicated that the genetic modifications in the original KO line modified Rapgap1 mRNA expression levels ( Figure 1B). *Strbp* mRNA expression levels were not changed when using multiple probes that were located downstream of the *GPR21* gene ( Figure 1A and data not shown). Also, *Rabgap1* mRNA expression levels were assessed using 2 Taqman probes ( Table 1) that amplified different regions of *Rabgap1* in liver and BAT of KO and their wildtype littermate mice. One primer/probe set spanned *Rabgap1* exon 3 and 4 ( Table 1), which is located upstream of the *Gpr21* gene. Another primer/probe set spanned *Rabgap1* exon 17 and 18, which is located downstream of the *Gpr21* gene ( Table 1). *Rabgap1* mRNA expression levels in liver and BAT of *Gpr21* KO mice were not changed compared to their wildtype littermate mice with the upstream primer/probe set ( Figure 1C), however it was dramatically decreased in liver and BAT of *Gpr21* KO mice compared to their wildtype littermate mice with the downstream primer/probe set ( Figure 1C).

**Table 1.** Sequences of the different probes used.

Primer/probe	Sequence (5’-3’) or product No
genotyping forward primer	CAGCATGAAGTGAGAGCCAG
genotyping reverse primer	CAAGTAGCCCAGTGCCAGAAG
*Gpr21* qPCR forward primer	CACCTGGGATGGTAATCAGAG
*Gpr2*1 qPCR reverse primer	TCACAATGATGTTGCCAGAAAT
*Gpr21* qPCR probe	FAM/TTCTGGCAC/Zen/TGGGCTACTTGGAAA/IABkFQ
*Rabgap1* primer/probe set 1	Applied Biosystems, Mm01327207_m1
*Rabgap1* primer/probe set 2	Applied Biosystems, Mm01327199_m1
*Strbp* primer/probe set	Applied Biosystems, Mm00486379_m1
microarray probe 1460486_PM	**Probe sequence (5’-3’)**
	GACAAAAGTTCGAGTGTGCTCACCT
	GTTCGAGTGTGCTCACCTAATGAAA
	GTGTGCTCACCTAATGAAAGGTTAT
	CCCTTCAGCAAACGAAGCACTACTG
	AGCACTACTGAAAACTTCTTTCTGA
	ATATGAAGTTGTGTGTTTGGAGAGT
	GAAAACCACAGCCAGTCCTTCAGTT
	TTCAGTTCGCCTGCCACAGTCTGGA
	GATAATGATGAACCTCTCTTGAGTG
	TGAACCTCTCTTGAGTGGATTTGGG
	GGGATGTATCCAAAGAATGTGCAGA
microarray probe 1443535_PM	**Probe sequence (5’-3’)**
	GAAATTAAAGCTATGTGACCACCCC
	AAACATTTCCATTCCATCTGTCAAA
	GGCTAAGAAGTTCCAGGGTTTCCTG
	CAGGGTTTCCTGCATTCCAAGAATG
	TTGTTTAACCCACAGAAGTTTTATG
	GTTTTATGTCATTTAGCCTGGTCTA
	AAAAGCTTGGGATCAGAACTGTTTC
	ATGGTTTTGTCTGTCTTGGTTTGAT
	TGTTGACTTATCAGTTAAACCACCA
	GAAACCTTAGGCTATTGCAAGACTT
	ATGCATACCTAGTTATTGCAGCTTC
microarray probe 1424188_PM	**Probe sequence (5’-3’)**
	GAAAGTCCCTACACACTGTAAAGTC
	TAAAGTCCTACTTTCCTGGCTGGAT
	GGCTGGATCTCTGTCAGGCCTCTGA
	CAGGTGTACATCTCACTGGTCAGGT
	GAAAATGGCAGTTTTAGCACCTTTT
	AGTGGTGTCACAAGTGGCTCATCCT
	CTCTGTGTGCAGGTAGCTTGGGTTT
	GTTGGCTTTTCTAATGCTTGATGAG
	TCTGTCTCGTTCAGTTAACCCAAAC
	AACCCAAACAGTATAAGCCCATCTT
	TGGACATTGTGTGCTAGGGTAGTTT

### Generation of *Gpr21* specific KO ( *Gpr21* TAL 29bp) mice

**Figure 2.**
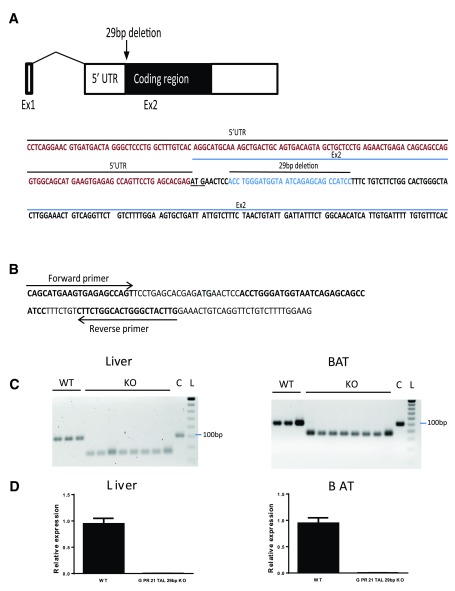
( **A**). Sequence and location of the 29 bp deletion in *Gpr21* TAL 29bp KO mice. ( **B**). sequence and location of genotyping primers. ( **C**). Genotyping of *Gpr21* TAL 29bp KO mice. Genomic DNA was generated from liver and BAT. PCR with genotyping primers amplified a 100 bp fragment from the genome of WT littermate mice and a 71 bp fragment from homozygous *Gpr21* TAL 29bp KO mice. C: commercial mouse genomic DNA. L: 20 bp DNA ladder. ( **D**). No wildtype *Gpr21* transcript were detected. qPCR analysis of *Gpr21* gene in liver and BAT using primer/probe set that located in the 29 bp region and only detect wildtype *Gpr21* transcript.

A new line of *Gpr21* KO mice ( *Gpr21* TAL 29bp) was created by deleting a 29 bp within the coding exon of *Gpr21* gene. Using the TALENS technology, a 29 bp very close to the ATG codon was deleted, thus causing an out of frame mutation and early termination of *Gpr21* gene ( Figure 2A). Homozygous *Gpr21* KO mice genotype was confirmed by PCR using primers located upstream and downstream of the 29bp deletion from genomic DNA of several tissues ( Figure 2B). As predicted, a 100 bp band was identified for the wildtype mice and a 71 bp band for the *Gpr21* TAL 29bp homozygous KO mice ( Figure 2C). The PCR fragment was sequenced and a 29 bp deletion was confirmed. From RNA isolated out of BAT and liver, no detectable *Gpr21* mRNA levels were identified in *Gpr21* TAL 29bp KO mice using qPCR and *Gpr21* probes that are located around the 29 bp deletion region ( Figure 2D). Similar mRNA levels were detected using primer/probe that were located downstream of 29bp deletion in exon 2 (data not shown). The result confirmed that *Gpr21* transcripts in *Gpr21* TAL 29bp KO mice had the 29bp deletion.

### *Rabgap1* and *Strbp* expression levels are not affected in *Gpr21* TAL 29bp KO mice

**Figure 3.**
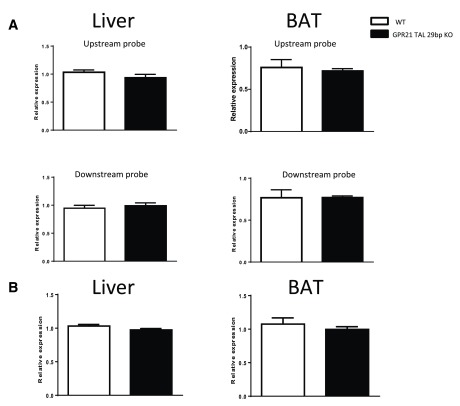
( **A**) *Rabgap1* mRNA expression levels were assessed using 2 Taqman probes in liver (top left panel) and BAT (top right panel) of GPR21 TAL 29 bp KO and their wildtype littermate mice. ( **B**) Liver (bottom left panel) and BAT (bottom right panel) strbp mRNA expression levels were assessed in wildtype and *Gpr21* TAL 29bp KO mice.

*Rabgap1* mRNA expression levels were assessed using 2 Taqman probes ( Table 1) that amplified different regions of *Rabgap1* in liver and BAT of KO and their wildtype littermate mice. One primer/probe set spanned *Rabgap1* exon 3 and 4 ( Table 1), which is located upstream of the *Gpr21* gene. Another primer/probe set spanned *Rabgap1* exon 17 and 18, which is located downstream of the *Gpr21* gene ( Table 1). *Rabgap1* mRNA expression levels in *Gpr21* TAL 29bp KO mice were not changed compared with their wildtype littermate mice with both primer/probe sets ( Figure 3A). Liver and BAT *Strbp* mRNA expression levels were also not changed between *Gpr21* TAL 29bp KO mice and their wildtype littermate mice ( Figure 3B). One of the limitations of our study is that we have not measured GPR21 protein levels.

### *Gpr21* TAL 29bp did not show improvements in glucose and insulin metabolism

**Figure 4.**
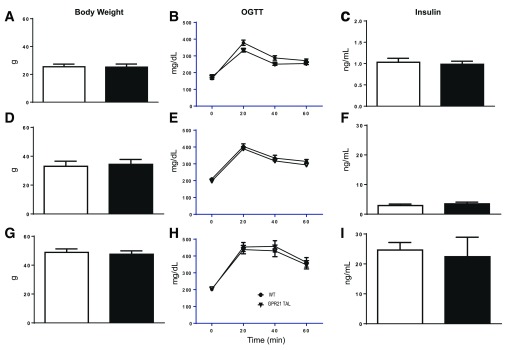
At 11 weeks of age, body weight (Figure **4A**), OGTT (Figure **4B**) and insulin levels (Figure **4C**) were measured in wildtype (open bar and open circle) and *Gpr21* TAL 29bp KO Mice (filled bar and open triangle) fed a normal chow diet. At 15 weeks of age and at 26 weeks of age body weight (Figure **4D** & **4G**), OGTT (Figure **4E** and **4H**) and insulin levels (Figure **4F** & **4I**) were measured in wildtype (open bar and open circle) and *Gpr21* TAL 29bp KO Mice (filled bar and open triangle) fed a high fat diet for 4 weeks and 15 weeks, respectively.

The body weight, OGTT and insulin levels of *Gpr21* TAL 29bp KO mice fed a normal chow were not different from the ones of their wildtype littermates ( Figure 4 A–C). Mice were then fed with a 45% high fat diet to induce obesity and insulin resistance. After 4 weeks and 15 weeks of high-fat feeding, *Gpr21* TAL 29bp KO mice gained similar body weight to that of their wildtype littermates, showed no difference on glucose tolerance and fasting blood glucose and insulin levels were not different from their wildtype littermates, Figures 4 D–I, respectively.

### Next Steps

The results of Osborn and Gardner suggest that GPR21 may play an important role in regulating body weight and glucose metabolism. However, in our attempts presented here to confirm their findings we didn’t see the same effect. We would therefore like to encourage an open discussion and collaborate with Osborn and Gardner as well as others in the wider community to further elucidate the potential effectiveness of pharmacologically inhibiting GPR21. In any work using genetically manipulated animal models, it is critical to demonstrate that the targeted manipulation behind the biological differences is being explored. While this should be obvious and generally assessed, this study illustrates one of the numerous ways in which scientists may be misled – changes in expression or function of other genes near the targeted gene. An analysis of the expression or function of nearby genes may be a general recommendation that could be made for all KO studies.
