# Supplementary material for: GPR21 KO mice demonstrate no resistance to high fat diet induced obesity or improved glucose tolerance
Source: F1000Res. 2016 Jun 17;5:136. Originally published 2016 Feb 4. [Version 2] doi: 10.12688/f1000research.7822.2 (PMC4813635; doi:10.12688/f1000research.7822.2)
Supplement: Peer review history

# Peer review: Referee response for version 2

**DOI:** 10.5256/f1000research.9484.r14428

**Published:** 2016-06-24

**Authors:** Mary Pelleymounter

**Affiliations:** 1 Office of Translational Research Program (OTR), National Institute of Neurological Disorders and Stroke (NINDS), Bethesda, MD, USA

In this first revision of the manuscript, “GPR21 KO mice demonstrate no resistance to high fat diet induced obesity or improved glucose tolerance”, the authors have addressed many of the concerns related to the addition of detail to the Materials and Methods section. However, the authors have not addressed the potential impact of differences in methodology between their physiological assessments and those of Gardner, *et al* and Osborne, *et al*. For example, they did not verify GPR21 KO in hypothalamic or brain tissue and they did not attempt to replicate the gender, diet or even OGTT methods from either study. Instead their methods were somewhat of a hybrid of the Gardner and Osborne studies and did not conduct any form of in-depth metabolic analysis. Therefore, although their data certainly does suggest that the Deltagen KO strategy did involve changes in Rabgap1 expression, it is difficult to claim that Rabgap1 reduction explained the phenotype of the Deltagen GPR21 KO mice if the physiological measures were not obtained in the same way as the two studies in question.   For these reasons, it would seem that the authors should discuss the caveats to their own study in the Next Steps section along with the suggestion to other investigators that GPR21 KO may not have been responsible for the metabolic phenotype observed by Gardner *et al* and Osborne, *et al*.

I have read this submission. I believe that I have an appropriate level of expertise to confirm that it is of an acceptable scientific standard, however I have significant reservations, as outlined above.

# Peer review: Referee response for version 1

**DOI:** 10.5256/f1000research.8421.r12685

**Published:** 2016-03-24

**Authors:** Michelle Kimple

**Affiliations:** 1 Division of Endocrinology, Diabetes and Metabolism, Department of Medicine, University of Wisconsin-Madison, Madison, WI, USA

The article by Wang and colleagues addresses the resistance of GPR21 knockout mice to insulin resistance and high-fat diet induced obesity and metabolic dysregulation. Two previous publications using a different GPR21 knockout mouse model generated with ES cells from Deltagen have been previously published: Osborn *et al*. (2012) JCI 122: 2444-2453; and Gardner *et al*. (2012) BBRC 418: 1-5. Osborn *et al*. demonstrated that, at a baseline of 8 weeks, GPR21 KO mice were significantly lighter than their wild-type controls despite similar food intake, and after 11 weeks on normal chow had significantly improved insulin sensitivity and glucose tolerance. In addition, GPR21 KO mice fed a 60 kcal% HFD for 12 weeks showed no differences in % weight gained or adiposity on the diet, but maintained a significantly improved glucose tolerance and insulin sensitivity. Gardner *et al*. demonstrated that, at baseline of 120 days, GPR21 KO mice were lighter, again despite similar food intake, and after 12 weeks on a 45 kcal% diet, gained significantly less weight and adiposity and maintained better insulin sensitivity and glucose tolerance than the wild-type controls. In contrast, Wang and colleagues find no differences in body weight, adiposity, or glucose and insulin tolerance between their GPR21 knockout mice (generated using TALEN technology) and wild-type controls; an important finding they suggest is due to unintentional changes in RabGAP1 gene expression in the original Deltagen GPR21 knockout mice.

**General Comments:**

1) The aim of the current work, as stated in the last paragraph of the introduction, was to show that the effects observed in Osborn and Gardner were not solely due to loss of GPR21. I believe the authors have essentially demonstrated this, and the title of the article is appropriate and reflective of this. Yet, much of the abstract, introduction and results sections focus on RabGAP1 expression. More confirmatory experiments seem necessary to implicate changes in RabGAP1 in the Deltagen GPR21 knockout phenotype (see specific comment 2). 

2) As a pure replicative paper, the authors did not directly replicate either of the original HFD studies performed in Osborn or Gardner. The 45 kcal% HFD protocol was closer to that described in Gardner, yet the mice were started on the HFD 6 weeks earlier (presumably at 11 weeks based on figure legends instead of ~17 weeks in Gardner) and continued on the diet 3 weeks longer. The statement in the "Next Steps" should be modified as such.  

**Specific Comments:**

1) In the Materials and Methods section, the description of the high-fat diet experiments are not detailed enough. This is important as the high fat diet experiments in the original referenced publications are actually quite different from each other. Furthermore, this would allow other investigators to more easily replicate the diet study.

2) The authors propose that changes in RabGAP1 expression might be responsible for the phenotypes observed in Gardner and Osborn. This conclusion would be stronger with qPCR analysis of the original Deltagen mouse tissues (such as in Figure 3, which was performed for the GPR21 TALEN KO mice) instead of reliance on microarray probe data. In such a case, a direct comparison of RabGAP1 expression could be made between the Deltagen and TALEN GPR21 knockout mice.

3) Hypothalamic GPR21 expression was proposed as responsible for phenotype of mice on normal chow observed in Osborn *et al*, and GPR21 is highly expressed in the hypothalamus. I would recommend adding this tissue to the panel analyzed by qPCR in Figure 3.

I have read this submission. I believe that I have an appropriate level of expertise to confirm that it is of an acceptable scientific standard, however I have significant reservations, as outlined above.

# Peer review: Referee response for version 1

**DOI:** 10.5256/f1000research.8421.r12759

**Published:** 2016-03-07

**Authors:** Richard Neubig

**Affiliations:** 1 Department of Pharmacology and Toxicology, Michigan State University, East Lansing, MI, USA

The title is appropriate.

The design, methods, and analysis are generally complete and appropriate with the following exceptions:

1. The methods to obtain data from the Deltagen GPR21 mutant mice are not sufficiently described. The source of the mice, genetic, background, and backcross history are not included in the methods. Since genetic background can profoundly change biological phenotypes, this information is essential to fully understand the differences between the Deltagen GPR21 KO and the new TALEN GPR21 KO mice described here.
2. The full sequence of the PCR product across the 29 bp deletion should be shown along with an alignment with the Gpr21 gene sequence to ensure that the deletion is indeed within the Gpr21 gene.
3. To definitively establish that the knockout does disrupt Gpr21, it would be helpful to additionally demonstrate reduced expression of the Gpr21 protein. This could be done by antibody detection methods, mass spec, or other functional assessments of Gpr21 activity.

 

Minor correction suggested:

1. Page 3 – “ *Strbp* … probes that were located upstream and downstream of the *Strbp* gene … “ should probably read “downstream of the *Gpr21* gene …”.

 

The conclusions require modification and they should also be expanded to reflect broader implications of this study.

1. The description of results in the “Next steps” paragraph should be amended. It is important in our discussions of “replication” that scientists be precise. *The term replication should be used when the precise methods and reagents were used for the follow-on studies.* This work does not “replicate” the original study but attempts to “confirm” or “extend” it using a different approach. Clearly robust scientific results must not only be replicable but also must be able to be confirmed or extended to have real value in advancing our understanding of biological results or drug action. I suggest the following. Replace “replicate their findings” with “confirm their findings using a Gpr21 KO mouse generated with a different technology”.
2. The limitation that the Gpr21 protein levels were not assessed should be included in the discussion unless data are shown to address that limitation.
3. It would also be valuable to note a more general conclusion from this study. In any work using genetically manipulated animal models, it is critical to demonstrate that the targeted manipulation is behind the biological differences being explored. While this should be obvious and generally assessed, this study illustrates one of the numerous ways in which scientists may be misled – changes in expression or function of other genes near the targeted gene. An analysis of the expression or function of nearby genes may be a general recommendation that could be made for all KO studies.

I have read this submission. I believe that I have an appropriate level of expertise to confirm that it is of an acceptable scientific standard.

# Peer review: Referee response for version 1

**DOI:** 10.5256/f1000research.8421.r12620

**Published:** 2016-03-07

**Authors:** Mary Pelleymounter

**Affiliations:** 1 Office of Translational Research Program (OTR), National Institute of Neurological Disorders and Stroke (NINDS), Bethesda, MD, USA

Dr. Wang and colleagues have provided intriguing evidence to suggest that the metabolic phenotype of the Deltagen GPR21 KO mouse may not be completely due to deletion of the GPR21 gene.  Rather, these authors suggest that the metabolic phenotype of the Deltagen GPR21 KO mouse could have been a function of the location of the 5.3 kb lacZ/Neo cassette within exon 1, which could have altered the expression and function of Rabgap1 and Strbp.  Indeed, the authors did show that Rabgap1 was altered in brown and white adipose tissue, liver and spleen of the Deltagen GPR21 KO mice.  In order to understand whether the metabolic phenotype of the Deltagen GPR21 KO mice was truly due to deletion of GPR21 or an artifact of the neo cassette location in the Deltagen mice, this group used a different strategy to delete GPR21 which did not involve Rabgap1 or Strbp.  The KO mice generated as a result of this alternate GPR21 deletion strategy (GPR21 TAL29bp KO) did not demonstrate the high fat diet resistance phenotype observed in the Deltagen GPR21 KO mice. In addition, while there were no differences in Rabgap1 mRNA or strbp mRNA in the liver or brown adipose tissue of the GPR21 TAL 29bp KO mice, there was clear deletion of GPR21 in the same tissues of these mice.

Although this information is quite important for the research area of obesity and diabetes therapeutics in general, there are some aspects of the experimental design and manuscript narrative that could be strengthened in order to provide more confidence in the findings.

1. It is unclear why the authors chose to focus on liver and brown adipose tissue expression levels of Rabgap1 and GPR21.  Tissue expression of GPR21 was much more prominent in brain and spleen in both the Gardner *et al*., and Osborn, *et al*., papers which described the high fat diet resistance phenotype in the Deltagen GPR21 KO mice.  It seems that there would be even more confidence in the findings of the current manuscript if GPR21 was as dramatically knocked out in brain and spleen by the GPR21 TAL29bp deletion strategy as it was in liver and BAT.
2. The argument that the improved metabolic phenotype in the Deltagen GPR21 KO mice was an artifact of neo cassette location would have been more convincing if the current authors had compared the Deltagen GPR21 KO mice in a  “head to head” fashion with the GPR21 TAL29bp KO mice when they evaluated resistance to diet-induced impairments in glucose tolerance.  This would have shown that the authors were able to reproduce the metabolic improvements previously observed in the high fat diet-fed Deltagen mice even though the GPR21 TAL29bpKO mice did not show such an improvement under the same experimental conditions.
3. Since it is important to assure that the experimental conditions utilized to assess metabolic phenotype in the current study were similar to those utilized for the Deltagen GPR21 KO mice in other labs, it would have been helpful if the authors had provided more detail about their methodology in the current manuscript.  For example, the following points are not clear from the narrative of the manuscript:
  a. What were the housing conditions of the animals (individual vs group-housed?, ambient temperature?
  b. Were the mice all one gender? (the Gardner paper used a mix of male and female, which could have influenced body weight, activity level, etc)
  c. What were the Ns for the OGTT, Insulin and BW data and how were they derived (basis for power analysis)?
  d. What statistical analysis strategy was used to evaluate the data?
  e. Was a baseline insulin level obtained under the conditions used for the OGTT?
  f. Were subsets of animals sacrificed after the respective OGTT and insulin measurements at each time point (11, 15 and 26 weeks)?  If so, how was that taken into account in the statistical analysis for data collected over the entire time period (i.e., body weight)?
4. In the “Next Steps” section, the authors suggest that the follow up to their data will be the responsibility of those that read their article.  However, their data only opens up the possibility that the metabolic phenotype of the Deltagen GPR21 KO mice is an artifact of neocassette location.  In this reviewer’s opinion, the authors still have quite a bit of work to do in order to provide definitive evidence that the Deltagen GPR21 KO mouse phenotype is not correct.  It would also be informative to hear the author’s view on the potential role of Rabgap1 in insulin sensitivity and resistance.

I have read this submission. I believe that I have an appropriate level of expertise to confirm that it is of an acceptable scientific standard, however I have significant reservations, as outlined above.
